# Validation of the 2^nd^ Generation Proteasome Inhibitor Oprozomib for Local Therapy of Pulmonary Fibrosis

**DOI:** 10.1371/journal.pone.0136188

**Published:** 2015-09-04

**Authors:** Nora Semren, Nunja C. Habel-Ungewitter, Isis E. Fernandez, Melanie Königshoff, Oliver Eickelberg, Tobias Stöger, Silke Meiners

**Affiliations:** Comprehensive Pneumology Center (CPC), University Hospital, Ludwig-Maximilians University, Helmholtz Zentrum München, Munich, Member of the German Center for Lung Research (DZL), Munich, Germany; University of Allabama at Birmingham, UNITED STATES

## Abstract

Proteasome inhibition has been shown to prevent development of fibrosis in several organs including the lung. However, effects of proteasome inhibitors on lung fibrosis are controversial and cytotoxic side effects of the overall inhibition of proteasomal protein degradation cannot be excluded. Therefore, we hypothesized that local lung-specific application of a novel, selective proteasome inhibitor, oprozomib (OZ), provides antifibrotic effects without systemic toxicity in a mouse model of lung fibrosis. Oprozomib was first tested on the human alveolar epithelial cancer cell line A549 and in primary mouse alveolar epithelial type II cells regarding its cytotoxic effects on alveolar epithelial cells and compared to the FDA approved proteasome inhibitor bortezomib (BZ). OZ was less toxic than BZ and provided high selectivity for the chymotrypsin-like active site of the proteasome. In primary mouse lung fibroblasts, OZ showed significant anti-fibrotic effects, i.e. reduction of collagen I and α smooth muscle actin expression, in the absence of cytotoxicity. When applied locally into the lungs of healthy mice via instillation, OZ was well tolerated and effectively reduced proteasome activity in the lungs. In bleomycin challenged mice, however, locally applied OZ resulted in accelerated weight loss and increased mortality of treated mice. Further, OZ failed to reduce fibrosis in these mice. While upon systemic application OZ was well tolerated in healthy mice, it rather augmented instead of attenuated fibrotic remodelling of the lung in bleomycin challenged mice. To conclude, low toxicity and antifibrotic effects of OZ in pulmonary fibroblasts could not be confirmed for pulmonary fibrosis of bleomycin-treated mice. In light of these data, the use of proteasome inhibitors as therapeutic agents for the treatment of fibrotic lung diseases should thus be considered with caution.

## Introduction

Idiopathic pulmonary fibrosis (IPF) is an irreversible, lethal fibrotic disease of the lungs. After diagnosis, the median survival is only up to 3.5 years due to its progressive nature, unspecific symptoms and therefore late diagnosis [1]. In IPF, excessive extracellular matrix deposition (ECM) within the fine alveolar structure leads to a gradual loss of elasticity which impairs proper gas exchange in the lungs and patients finally die of lung failure [2,3]. Despite major progress in the last years, therapeutic interventions in IPF are still very limited [4–6]. In most cases, lung transplantation remains the only option. Currently, there is only one drug, pirfenidone, a small molecule with antifibrotic and anti-inflammatory properties, approved in Europe for the treatment of IPF [4–6]. The pathomechanism of IPF is not fully understood yet, but it is proposed that repeated microinjuries of epithelial cells induce a wound healing response during which fibroblasts differentiate into myofibroblasts. These activated myofibroblasts express α smooth muscle actin (αSMA) and release ECM proteins like collagens and fibronectin to promote matrix deposition and tissue remodelling. Under physiological conditions, the remodelling process stops once wound healing is finished. In lungs of IPF patients, myofibroblasts remain active and deposit excessive amounts of ECM. This leads to a destruction of alveolar organisation, loss of elastic recoil of the lung and the rapid decrease of lung function in patients. TGF-β has been identified as a main profibrotic cytokine involved in myofibroblast differentiation and as a driving factor for pathogenic pulmonary fibrosis [3,7].

The ubiquitin proteasome system (UPS) is responsible for the controlled degradation of most intracellular proteins [8]. Proteins are targeted for degradation by the proteasome by linkage to polyubiquitin chains as a degradation signal to be processed by the proteasome [9]. Polyubiquitination proceeds along a cascade of enzymatic reactions involving E1, E2 and E3 enzymes which transfer activated ubiquitin to a lysine residue of the substrate protein. Polyubiquitinated proteins are then transferred to and hydrolyzed by the proteasome. The proteasome is a multicatalytic enzyme complex. It consists of a barrel-like structured catalytic core particle, also named 20S proteasome, which contains three active sites residing in the β5, β2, and β1 subunits that cleave polypeptides after different amino acids. Therefore, they are named chymotrypsin-like (CT-L), trypsin-like (T-L), and caspase-like (C-L) active sites, respectively. For optimal activity, the 20S proteasome has to be attached to a regulatory particle, the 19S complex, which is the most abundant proteasome regulator to catalyze ubiquitin-dependent protein degradation [10]. The 20S core particle and 19S regulator together build the 26S proteasome. The 19S regulatory complex is responsible for recognition of polyubiquitinated substrates, deubiquitination, and ATP-dependent protein unfolding and translocation of proteins into the 20S catalytic core [11]. A variety of proteasome inhibitors have been designed to covalently bind or reversibly interact with the N-terminal threonine residue that forms the active site of the β1, β2, and β5 subunits [9,12]. Bortezomib is the first FDA approved proteasome inhibitor and registered for the treatment of multiple myeloma and relapsed and refractory mantle cell lymphoma. It is a dipeptidyl boronic acid and has high binding specificity to the CT-L and C-L active sites [13,14]. In the past years, several second generation proteasome inhibitors have been developed to provide higher selectivity for specific active sites [15]. Just recently, the CT-L specific inhibitor carfilzomib has been FDA-approved for the treatment of multiple myeloma. It irreversibly and selectively binds to the CT-L active site by formation of a morpholine ring with the N-terminal threonine within the catalytic core particle [16]. Oprozomib (former ONX0912) is a novel modified derivate of carfilzomib bearing the same epoxyketone pharmacophore. Oprozomib is the first orally available proteasome inhibitor [17,18].

Inhibition of the proteasome has been shown to provide antifibrotic effects in different tissues and several experimental mouse models [19–21]. The mechanism by which these antifibrotic effects are mediated are not fully unravelled but appear to involve attenuation of profibrotic TGF-β signalling [19,21]. Even though results obtained *in vitro* with fibroblasts from different tissues are quite promising, animal studies of bleomycin induced lung fibrosis are controversial [22]. Mutlu et al. could prevent lung fibrosis in the bleomycin mouse model by intraperitoneal (ip) application of 0.12 mg/kg body weight bortezomib twice at day 7 and 14 with sacrificing of the mice at day 21 after bleomycin challenge [21]. In contrast, Fineschi et al observed no therapeutic effects, but twice weekly intravenous application of 0.8 mg/kg bortezomib between day 7 and 21 induced early death of all mice whereas in the non-bleomycin control group this bortezomib treatment scheme was well tolerated [23,24]. As bortezomib doses applied by Fineschi et al. were quite high and also given more often compared to the study conducted by Mutlu et al., this might have caused detrimental side effects in the bleomycin-injured lungs. These data accord well with the established concept that the degree of proteasome inhibition in a given cell determines the biological outcome ranging from beneficial to cytotoxic effects [23,24].

Due to the observed systemic cytotoxic side-effects of bortezomib in animal studies [23] we hypothesized that local application of low doses of a more specific and less toxic second generation proteasome inhibitor, i.e. oprozomib, into the lungs will be better tolerated as systemic side-effects are reduced but may efficiently mediate antifibrotic effects in experimental pulmonary fibrosis.

## Materials and Methods

All animal experiments were conducted according to international guidelines and were approved by the local administrative government Regierung Oberbayern (55.2–1–54–2531–86–11).

### Isolation and cell culture of murine lung fibroblasts and alveolar epithelial type II cells

Female FVB.129S6-Gt(ROSA)26Sor^tm2(HIF1A/luc)Kael^/J mice (Stock Number: 006206, The Jackson Laboratory), containing the ODD-Luc transgene, were dissected to obtain whole lungs. Isolated lungs were collected in pre-warmed DMEM/F-12 (Gibco) supplemented 1% penicillin/streptomycin (Gibco) and 20% FBS (PAA) and cut into pieces. Lung pieces were placed into 50 ml falcon tubes and digested at 37°C for 2h, using Collagenase 1 (Biochrome). Digested tissue was then filtered through a 70 μm nylon filter for further mincing. After washing and pelleting minced tissue was resuspended in supplemented DMEM/F-12 and incubated at 37°C at 95% humidity and 5% CO2. Media was changed every three days. Primary murine lung fibroblasts (pmLF) were split for the first time after reaching confluency of 80–90%. Cells were used up to passage three after first splitting. Cells were treated with proteasome inhibitor in DMEM/F-12 medium supplemented with penicillin/streptomycin and 20% FBS for up to 72 hours. Primary mouse alveolar epithelial type II (mATII) cells were isolated from C57BL/6 mice according to the protocol described by Königshoff et al. [25].

### Cell culture

The A549 human alveolar epithelial cell line was obtained from ATCC (American Type Culture Collection). A549 cells were cultured in DMEM media (Gibco). Media was supplemented with 10% FBS. Cells were kept at 37°C and 95% humidity containing 5% CO_2_.

### MTT Assay

Cytotoxicity of oprozomib and bortezomib was assayed by using the 2.5-diphenyltetrazolium bromide (MTT) assay. Briefly, 5 × 10^4^ cells (A549, pmLF) per well were seeded in 24-well plates or 2.5 × 10^4^ cells (mATII cells) were seeded in 48 well plates. The next day, cells were incubated with different concentrations of oprozomib or bortezomib for 72 hours (A549, pmLF) or 52 hours (mATII cells). Then, a solution of 5 mg thiazolyl blue tetrazolium bromide (Sigma) per milliliter PBS was added to each well and incubated at 37°C for 1 hour to allow reduction of the tetrazolium dye to its insoluble formazan within the cell. After aspiration of the supernatant, the blue formazan crystals were dissolved in isopropanol + 0.1% Triton X-100 (Life Technologies). Absorbance was measured at 570 nm in a Tristar LB 941 plate reader (Berthold Technologies).

### Proteasome activity assay

Chymotrypsin-like and caspase like proteasome activities were determined in cell and tissue lysates using specific luminogenic substrates (Suc-LLVY-aminoluciferin and Z-nLPnLD-aminoluciferin, respectively) (Proteasome-Glo Assay System, Promega) according to the manufacturer’s instructions. Cells were harvested by trypsinization and cell pellets were snap-frozen in liquid nitrogen. Mouse and human lung tissue samples were homogenized using a Micro-Dismembrator (Sartorius). Cells or tissue were suspended in Milli-Q water (Millipore) containing protease inhibitor cocktail (cOmplete, Roche) and phosphatase inhibitor (PhosStop, Roche). Hypoosmotic lysis was performed by repeated freeze and thaw cycles. Cell debris and non-soluble fractions were removed by centrifugation (15000 g, 4°C, 30 min) and supernatants were collected to measure protein concentrations (Pierce BCA kit, Thermo Fisher) and proteasome activities. To measure proteasome activities, 3 μg of protein were diluted in Milli-Q water and added to the assay buffer. Luminescence was detected using a TristarLB 941 plate reader. Data are shown as relative values to the activity of untreated controls of the same experiment. In cell culture experiments, the control of each experiment and in animal experiments the average value of control groups is set as one.

### Luciferase Assay

Primary murine lung fibroblast were seeded at a density of 5000 cells per 0.32 cm^2^ (96 well plate) in culture medium and treated with different concentrations of oprozomib the next day. 24 hours later luciferase activity was assayed using the Bright-Glo system (Promega) according to the manufacturer’s protocol. Briefly cells were lysed in 50 μl of Glo-Lysis buffer (Promega). 20 μl of cell lysate was transferred into a white walled 96 well plate and 20 μl of Bright-Glo-Luciferase was added to each well. Luminescence was measured immediately using a Tristar LB 941 plate reader.

### Native gel analysis

Native gel electrophoresis was performed using the XCell SureLock Mini-Cell system (Life Technologies). 40 μg of protein per sample from hypoosmotic lysates were loaded on 10% nondenaturing gels and run for 4 h at 110 V and 4°C. Native gels were soaked with luciferase assay buffer containing 1 mM D-luciferase (Sigma) 25mM glycylglycine, 15 mM potassium phosphate, 15 mM MgSO_4_, 4mM EGTA, 2 mM ATP, 1 mM DTT, and 2 mM ATP and imaged for 30 min in the ChemiDoc XRS+ system (Bio-Rad). Afterwards, native gels were washed and incubated for 30 min at 37°C in proteasome activity assay buffer (50 mM Tris, pH 7.5, 10 mM MgCl_2_, 1 mM ATP, 1 mM DTT) containing 50 μM Suc-LVVY-AMC (Bachem), a fluorogenic, synthetic peptide substrate of the chymotrypsin-like activity of the proteasome. Gels were imaged at excitation wavelength of 380 nm and emission wavelength of 460 nm in the ChemiDoc XRS+ system.

### Immunofluorescence staining

Primary murine lung fibroblasts were cultured at a density of 5000 cells per 0.32 cm^2^ (96 well plate) in DMEM/F-12 supplemented with 1% penicillin/streptomycin and 20% FBS. The next day, cells were incubated with 50 nM or 100 nM of oprozomib or DMSO as control or treated with 5 ng/ml of TGF-β1 (R&D Systems) and incubated with 50 nM of oprozomib after 24 hours for an additional 24 hours. Cells were then washed with PBS and fixed with 4% paraformaldehyde (PFA), washed again and permeabilized with 0.25% Triton X-100. After further washing, primary antibody for Collagen type I (Coll-I) (Rockland) was added at a dilution of 1:200 for 1 hour. Secondary antibody Alexa Fluor 488 (Life Technologies) was added after washing and incubated for additional 45 minutes in darkness. Phalloidin Alexa Fluor 568 (Life Technologies) and DAPI staining (Sigma) was performed and finally cells were fixed with 4% PFA and stored at 4°C in the dark. Imaging was performed by fluorescent microscopy (LSM710 System, Carl Zeiss).

### BrdU cell proliferation assay

A colorimetric BrdU cell proliferation assay (Roche) was performed according to the manufacturer’s protocol. Briefly, primary murine lung fibroblasts were plated at a density of 5000 cells per 0.32 cm^2^ (96 well plate) in culture medium and treated with 10 nM, 50 nM, or 100 nM of oprozomib the next day for 72 hours. Cells were then labelled for 2–4 hours using 10 μM BrdU, dried at room temperature and stored at 4°C overnight. The next day, cells were fixed and DNA was denatured by adding FixDenat and incubated with anti-BrdU-POD antibody for 90 minutes at room temperature. Cells were washed and substrate solution was added. The reaction was stopped by adding H_2_SO_4_ to a final concentration of 0.2 M. Absorbance measurement was performed within 5 minutes at 450 nm using a Tristar LB 941 plate reader.

### RNA preparation and qRT PCR analysis

Total RNA was prepared from cells using the Roti-Quick-Kit according to manufacturer’s recommendations (Carl Roth) and transcription of 500 ng was performed using random hexamers (Life Technologies) and M-MLV reverse transcriptase (Sigma). For quantitative PCR reactions, we used the SYBR Green LC480 System (Roche). mRNA expression was normalized to the hypoxanthine phosphoribosyl transferase (Hprt) gene as a housekeeping gene (primer sequences available on request) (dCT) and further normalized to the mean value of the control group (ddCT).

### Animal experiments

For initial dose finding pathogen-free female FVB wildtype mice were obtained from Jackson Laboratory. Oprozomib was suspended in a solution of 0.1% Pluronic F-127 (Calbiochem, Merck Millipore) in Milli-Q water and applied once via intratracheal instillation. For validation of antifibrotic effects in the reporter mouse FVB.129S6-Gt(ROSA)26Sor^tm2(HIF1A/luc)Kael^/J mice were obtained from Jackson Laboratory. Pulmonary fibrosis was induced as described below and oprozomib was applied via intratracheal instillation as described for FVB wildtype mice. Pathogen-free female C57BL/6 mice (10–12-weeks old) were obtained from Charles River to validate antifibrotic effects of oprozomib after oral application. All mice were housed in rooms maintained at constant temperature and humidity with a 12 hours light cycle. Animals were allowed food and water ad libitum. Mice were sacrificed under ketamine/xylazine anesthesia after lung function measurement by exsanguination, and tissue processing was performed as previously described [26]. Pulmonary fibrosis was initiated by a single intratracheal instillation of 50 μl of bleomycin (3 U/kg, Sigma), dissolved in sterile saline, using the MicroSprayer Aerosolizer, Model IA-1C (Penn-Century) as published before [27]. Control mice were instilled with 50 μl of saline. According to the treatment protocol, oprozomib was either instillated intratracheally into the lungs or applied orally using a gavage needle. For intratracheal instillation of bleomycin or oprozomib animals were narcotized by i.p. administration of medetomidin/midazolam/fentanyl. After instillation narcosis was antagonized by s.c. injection of atipamezole/flumazenile/naloxone. The experiment was aborted and euthanasia was performed, when animals showed the following signs of a poor general condition: weight loss >15%, scrubby fur, or bent back. No animal died without euthanasia. Animals were looked after every day after bleomycin challenge to monitor their general condition. In total 6 animals were euthanized by cervical dislocation due to strong weight loss >15%. Lungs were lavaged and perfused with PBS. Right lungs were snap frozen in liquid nitrogen for further analysis of RNA and protein and left lungs were infused with 4% PFA via the left main bronchus and submerged in 4% PFA for at least 24 hours and processed for paraffin embedding. Sections of 3 μm were cut, mounted on glass slides and stored at 4°C until further preparation.

### Histological analysis

Lung sections were incubated at 60°C at least for 30 minutes before H&E staining was performed. Sections were deparaffinized and transferred in water and hematoxylin and eosin staining was performed according to the manufacturer’s protocol. Briefly, slices were incubated in Hemalaun staining for 6 minutes (Carl Roth), washed and transferred into Eosin G (Carl Roth) 0.5% containing one drop of acidic acid per 100 ml for 10 minutes. Sections were washed, dehydrated in ethanol and covered using Entellan (Millipore) as cover medium. For imaging, sections were scanned in using a MiraxScan (Zeiss) and analysed with the Panoramic Viewer software (3DHISTECH). For immunofluorescence staining, paraffin embedded tissue slides were rehydrated and antigen retrieval was performed in citrate buffer (pH 6.0) for 30 seconds at 125°C and 10 seconds at 90°C. Slides were blocked with 5% BSA to avoid unspecific antibody binding and primary antibodies against Coll-I (Rockland) and αSMA (Sigma) were applied. Slides were washed with Tris buffer and incubated with secondary antibodies Alexa Fluor 568 anti-rabbit (Coll-I) and Alexa Fluor 488 anti-mouse (αSMA) for one hour in darkness. Counterstaining with DAPI was performed and slides were covered using Fluorescent Mounting Medium (DAKO).

### Statistics

Data are presented as means ± SEMs as indicated and were considered statistically significant when *P* ≤ 0.05. Statistical analysis was performed using 1way ANOVA, followed by Post-hoc testing for multiple comparisons (Bonferroni-Holmes or Dunnett’s test), Mann-Whitney t-test, or paired t-test.

## Results

### Specific inhibition of the CT-L active site by oprozomib is less toxic compared to bortezomib in alveolar epithelial cells

Local pulmonary drug application exposes lung epithelial cells to high drug concentrations as they constitute the first cellular barrier for the lung towards the environment [28]. Any local treatment strategy for lung fibrosis should thus provide antifibrotic effects in lung fibroblasts while maintaining pulmonary epithelial integrity. Therefore, we first analyzed the cytotoxicity and proteasome inhibition profile of the novel second generation inhibitor oprozomib (OZ) and compared it to the FDA-approved proteasome inhibitor bortezomib (BZ) in the human alveolar adenocarcinoma cell line A549 and in primary murine alveolar epithelial type II cells (mATII).

**Fig 1:**
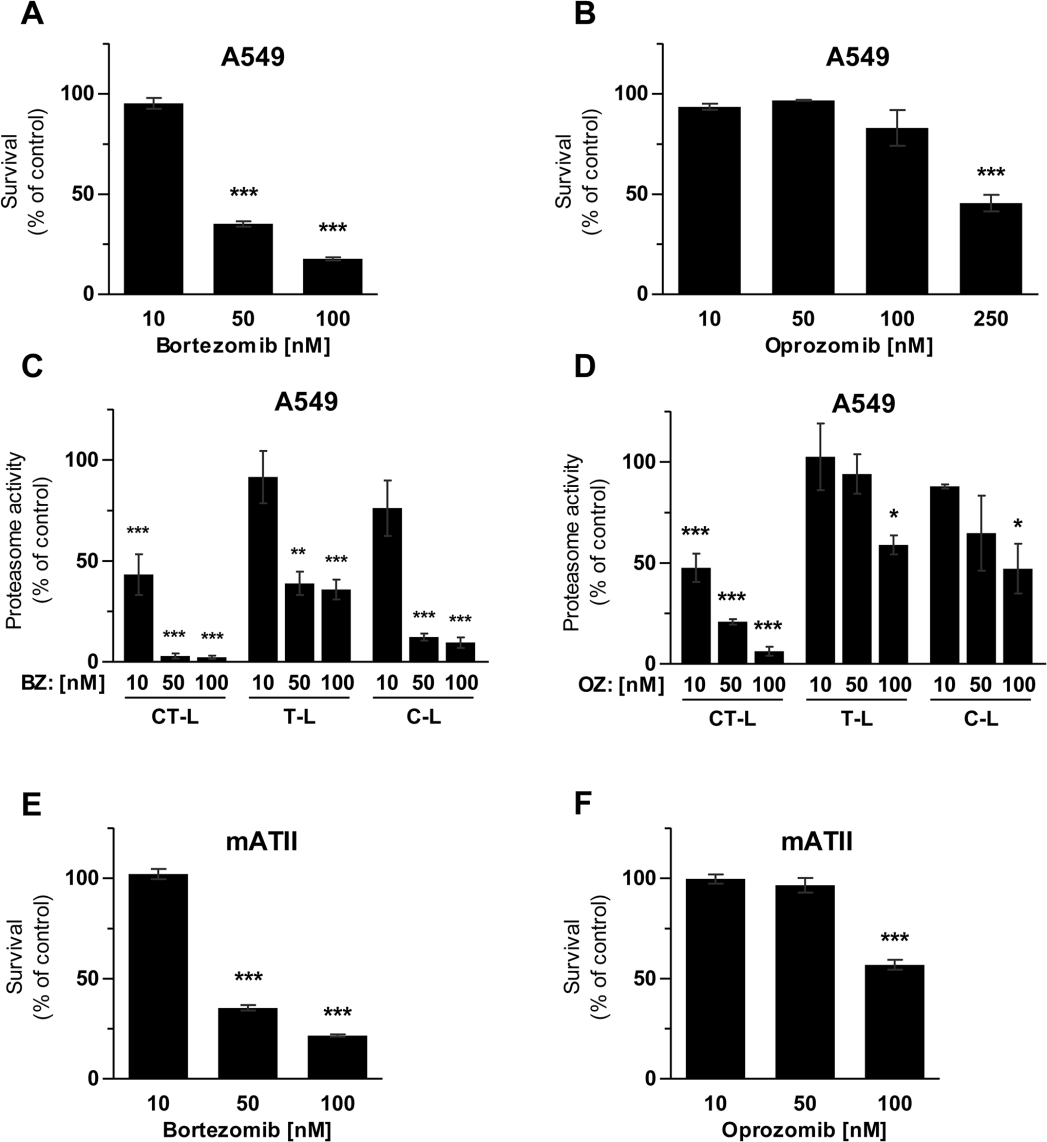
Toxicity and inhibitory profile of bortezomib and oprozomib in alveolar epithelial cells. MTT assay after 72 hours of treatment with (A) BZ or (B) OZ (Data represent mean ± SEM. *n* = 3 per group. *P ≤ 0.05, **P < 0.01, ***P < 0.001. 1way ANOVA Dunnett‘s Multiple Comparison Test). (C) Proteasome activity 24 hours after treatment with BZ or (D) OZ (Data represent mean ± SEM. *n* = 3 per group. *P ≤ 0.05, **P < 0.01, ***P < 0.001. 1way ANOVA Dunnett‘s Multiple Comparison Test). (E) and (F) MTT assay of primary murine ATII cells after 52 hours of treatment with OZ or BZ (Data represent mean ± SEM. *n* = 4 per group. *P ≤ 0.05, **P < 0.01, ***P < 0.001. 1way ANOVA Dunnett‘s Multiple Comparison Test).

Treatment of A549 cells with doses of 10 to 100 nM OZ for 72 hours was well tolerated, while 250 nM OZ caused pronounced loss of cell viability as assessed by MTT assays (Fig 1A). In contrast, BZ doses of more than 10 nM induced severe cytotoxicity by about 60% (for 50 nM) and 75% (for 100 nM) (Fig 1B). The less toxic effects of OZ correlated well with a high selectivity of OZ towards the chymotrypsin-like (CT-L) active site of the proteasome compared to BZ: after 24 hours of inhibitor treatment, 10 and 50 nM of OZ specifically inhibited only the CT-L active site, while the trypsin-like (T-L) and caspase-like (C-L) activities were only marginally affected (Fig 1C). The non-toxic dose of BZ, i.e.10 nM, also specifically inhibited the CT-L active site only, while higher doses of 50 and 100 nM also significantly blocked the T-L and C-L active sites (Fig 1D). The increased CT-L-specificity of oprozomib thus provides reduced toxicity in alveolar epithelial like cells compared to bortezomib. To confirm lower toxicity of OZ compared to BZ in a more physiological setting, we isolated murine ATII cells and treated them for 52 hours with different doses of OZ or BZ. While 50 nM of OZ was well tolerated by the mATII cells 50 nM of BZ reduced survival by up to 35%. These findings were well in accordance with our results from the A549 cells.

### Oprozomib specifically inhibits the chymotrypsin like active site of the 20S proteasome at non-toxic doses in primary mouse lung fibroblasts

To evaluate the inhibitory profile and toxicity of OZ in the main effector cells of pulmonary fibrosis, i.e. lung fibroblasts, we treated primary mouse lung fibroblasts with different concentrations of OZ for up to 72 hours. These fibroblasts were isolated from mice containing the ODD-luc reporter for proteasome inhibition. In the ODD-luc mouse model, an oxygen-dependent degradation domain (ODD) is fused to luciferase (luc), which serves as a proteasomal degradation signal for the luciferase fusion protein [29,30]. The ODD domain is derived from the hypoxia inducible factor (HIF)-1α and allows for proteasomal degradation of the HIF-1α transcription factor under physiological oxygen conditions. Under hypoxic conditions, however, ODD is hydroxylated and stabilizes HIF-1α thereby activating a protective gene program to counteract hypoxia [31]. The ODD-luc reporter thus accumulates at hypoxic conditions but also after inhibition of the proteasome and has thus been established to quantitatively monitor inhibition of the proteasome in cells and mice [32].

**Fig 2:**
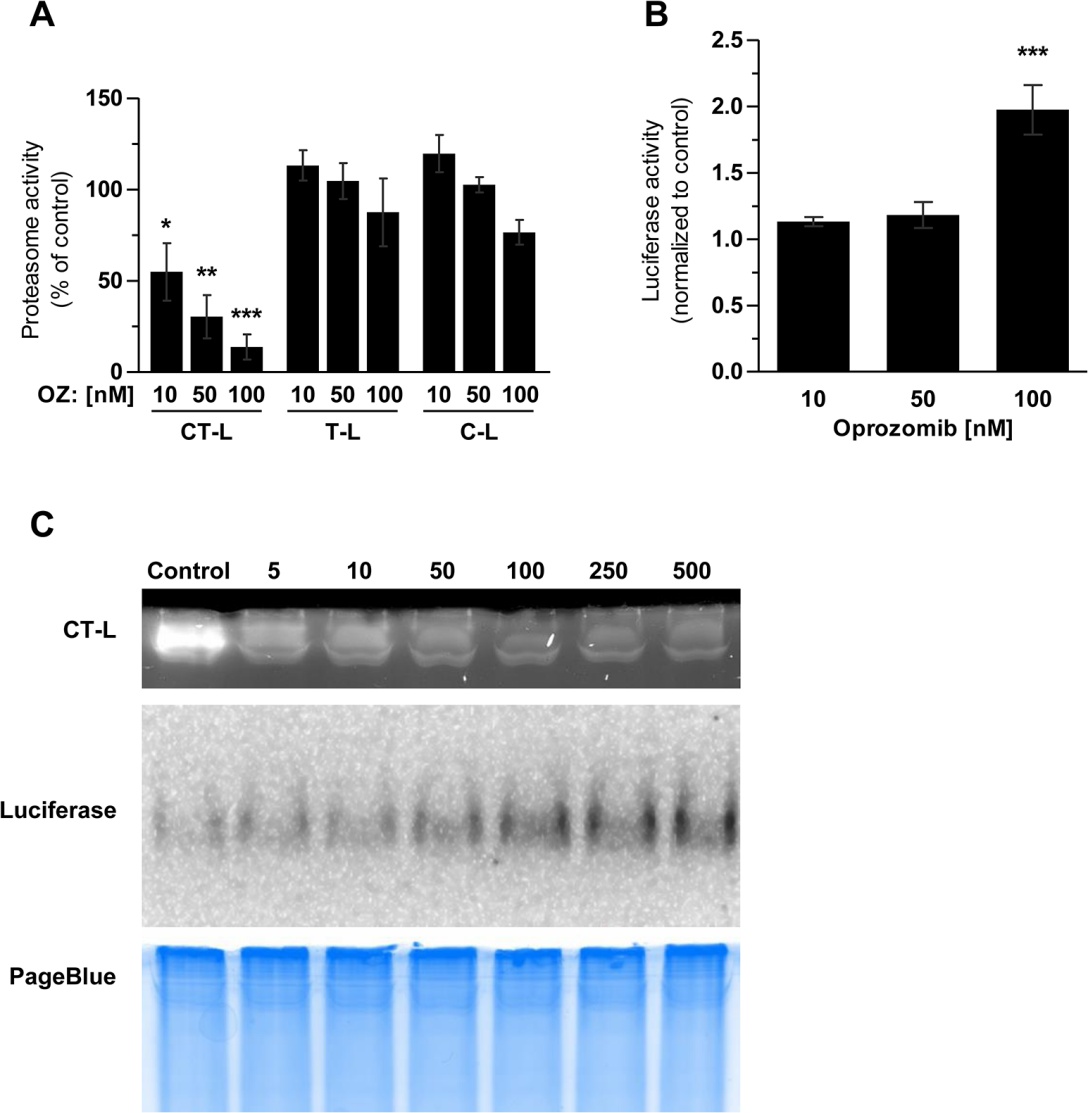
Inhibition profile of oprozomib in primary mouse lung fibroblasts. (A) Proteasome activity and (B) Luciferase activity of ODD-Luc FVB-LF 24 hours after treatment with OZ (Data represent mean ± SEM. *n* = 3 per group. *P ≤ 0.05, **P < 0.01, ***P < 0.001. 1way ANOVA Dunnett‘s Multiple Comparison Test). (C) Native gel of ODD-Luc FVB-LF 24 hours after OZ treatment.

In primary lung fibroblasts of the ODD-luc reporter mice, we observed a significant and specific reduction of the CT-L activity of the proteasome already at a concentration of 10 nM whereas T-L and C-L activities were not affected after 24 hours of treatment with OZ. Higher doses of 100 nM OZ inhibited the CT-L activity by 85% but only marginally affected the other two active centers (Fig 2A). Accumulation of the luciferase reporter was observed only at a dose of 100 nM OZ as measured by an increase in luciferase activity in the same cell extracts (Fig 2B). We further applied native gel analysis to directly visualize the dose dependent inhibition of the proteasome and accumulation of luciferase in a single experiment (Fig 2C). Overlay of the native gel with a CT-L specific fluorogenic substrate and luciferin showed a dose dependent reduction in proteasome activity from 10 to 500 nM OZ and a corresponding dose dependent increase in luciferase expression starting at 50 nM OZ and clearly visible from 100 nM onwards (Fig 2C). These experiments clearly show that OZ-mediated specific inhibition of the proteasomal CT-L activity by 70–90% results in the accumulation of a proteasomal reporter protein. Importantly, a dose of 50 nM OZ is still non-toxic. However, viability of primary mouse lung fibroblasts was reduced after 72 hours treatment with 100 nM OZ (S1 Fig).

### Oprozomib provides antifibrotic effects in primary lung fibroblasts

**Fig 3:**
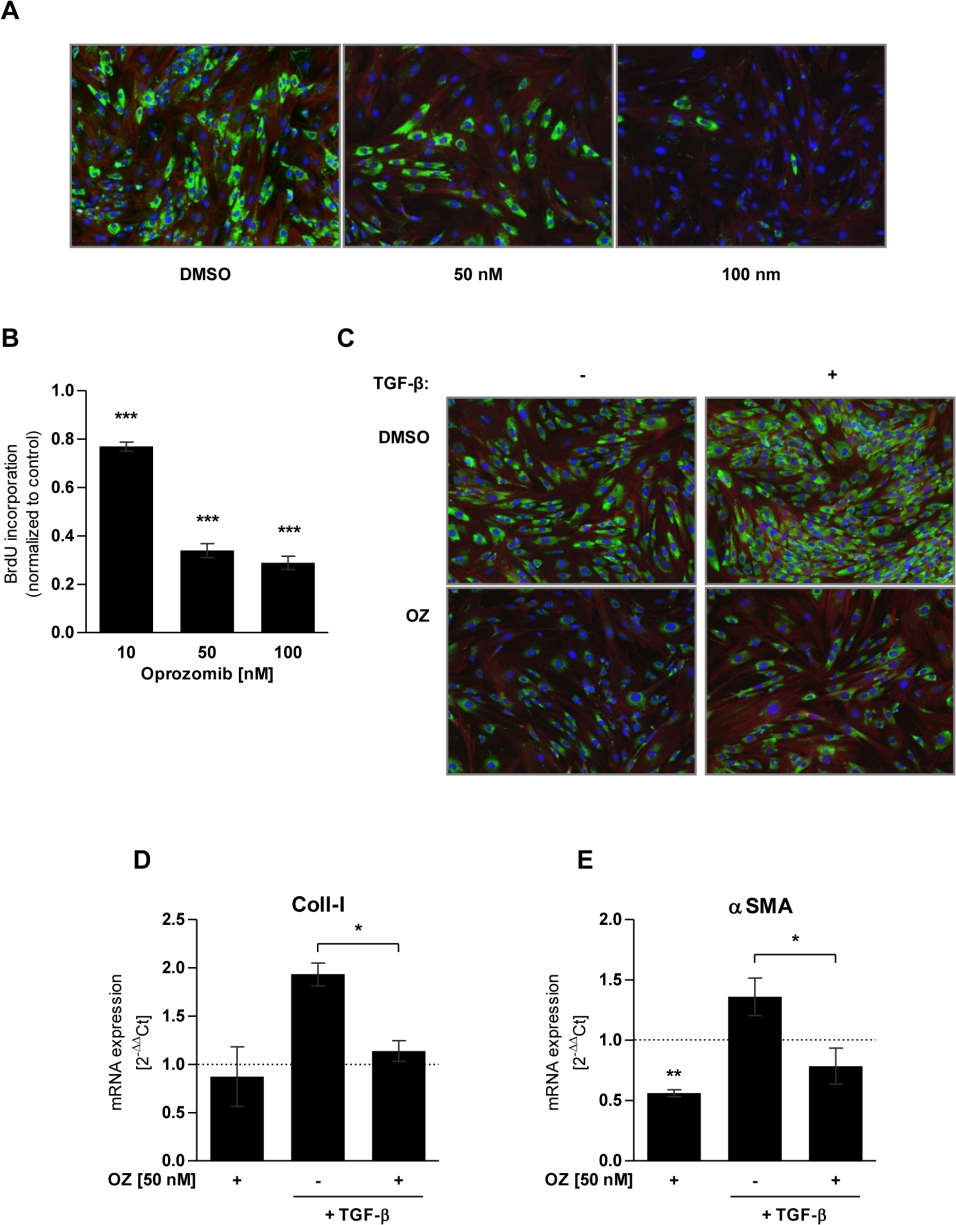
Antifibrotic effects of oprozomib in primary mouse lung fibroblasts. (A) Immunofluorescence staining for Coll-I (green), F-Actin (red) and nuclei (blue) after 72 hours of treatment with OZ. (B) BrdU proliferation assay of primary lung fibroblasts treated with OZ for 72 hours (Data represent mean ± SEM. *n* = 4 per group. *P ≤ 0.05, **P < 0.01, ***P < 0.001. 1way ANOVA Dunnett‘s Multiple Comparison Test). (C) Immunofluorescence staining for Coll-I (green), F-Actin (red) and nuclei (blue) after treatment with TGF-β and OZ. (D) and (E) RT-qPCR analysis of mRNA expression of Coll-I and αSMA after treatment with TGF-β and OZ (Data represent mean ± SEM. *n* = 3 per group. *P ≤ 0.05, **P < 0.01, ***P < 0.001. Paired t-Test).

In a next step, we evaluated whether the observed site-specific and non-toxic inhibition of the proteasome by OZ also provided antifibrotic effects in lung fibroblasts. Primary mouse lung fibroblasts were treated with 50 nM or 100 nM of OZ for 72 hours and expression of the profibrotic marker Coll-I was determined. We observed pronounced reduction of Coll-I expression as shown by immunofluorescence staining of the cells (Fig 3A). Further, BrdU incorporation and therefore proliferation were significantly reduced in OZ treated fibroblasts in comparison to DMSO treated control cells (Fig 3B).

As Mutlu et al identified impaired TGF-β signalling upon proteasome inhibition by BZ as a possible mechanism for the antifibrotic effects after proteasome inhibition [21], we tested if OZ also counteracts profibrotic TGF-β signalling. For that, we first treated primary mouse lung fibroblasts with TGF-β (5 ng/ml) for 24 hours, added 50 nM of OZ and incubated the cells for another 24 hours for immunofluorescence staining and 72 hours for RNA expression analysis. OZ treatment of mouse lung fibroblasts efficiently inhibited TGF-β induced Coll-I expression, both on the protein and mRNA level (Fig 3C and 3D). In addition, mRNA expression of the myofibroblasts marker αSMA was also significantly reduced in OZ treated cells. Of note, αSMA mRNA levels were already reduced by OZ treatment in the absence of TGF-β stimulation (Fig 3E). These data indicate that OZ efficiently counteracts TGF-β-mediated profibrotic responses by transcriptional downregulation of myogenic marker genes. Taking together, these *in vitro* data indicate that OZ represents a novel second generation proteasome inhibitor that specifically inhibits the chymotrypsin-like active site of the proteasome and thereby confers antiproliferative and antifibrotic effects on lung fibroblasts in the absence of major cytotoxic side effects on fibroblast and alveolar epithelial cells.

### Local pulmonary application of oprozomib efficiently inhibits proteasome activity in whole lung tissue at non-toxic concentration

**Fig 4:**
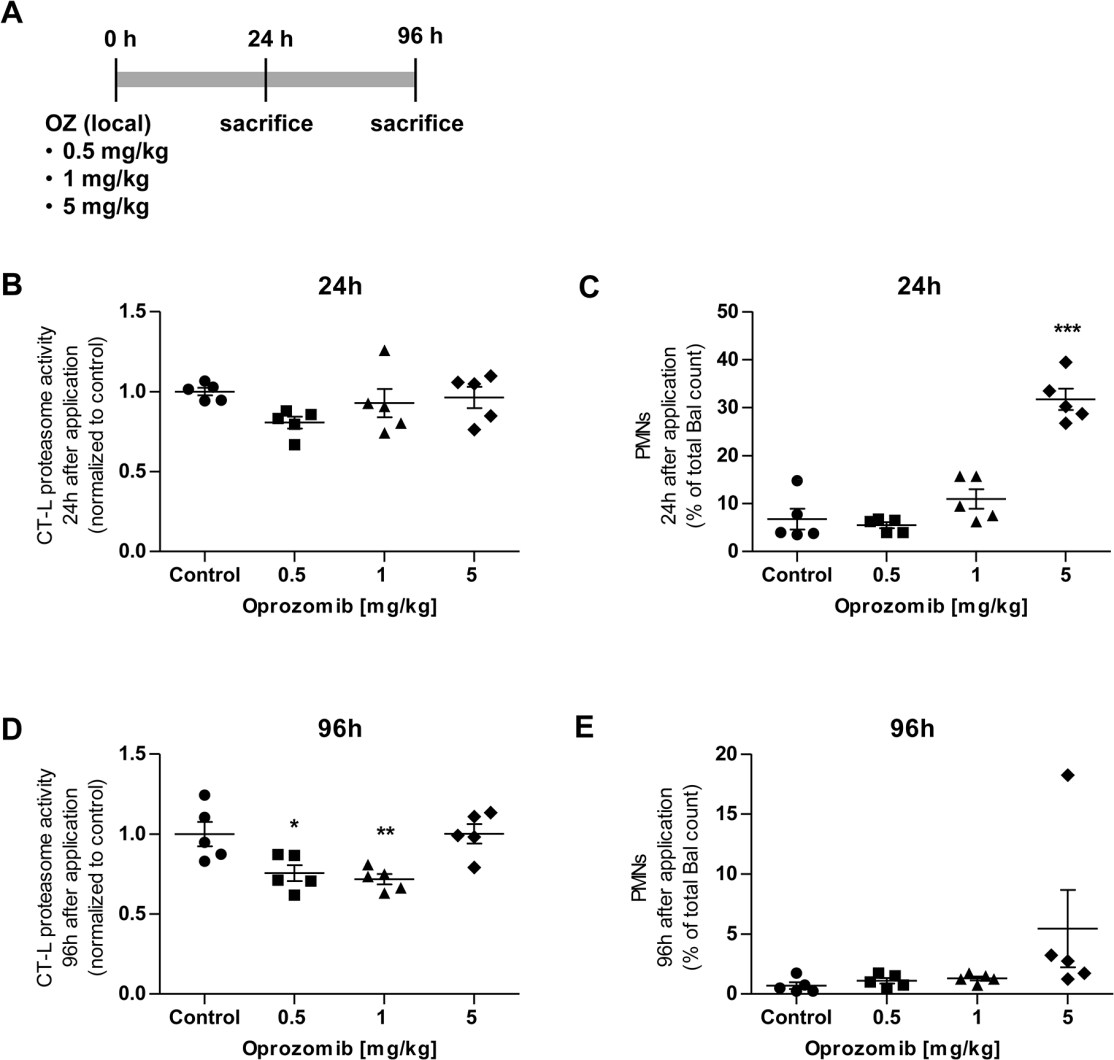
Dose response to local pulmonary application of 0.5, 1, and 5 mg/kg OZ or Pluronic F-127 0.1% solvent control after 24 hours or 96 hours. (A) Treatment scheme: local pulmonary application of OZ, (B) CT-L proteasome activity after 24 hours, (C) percent of PMNs to total BAL count after 24 hours, (D) CT-L proteasome activity after 96 hours, and (E) percent of PMNs to total BAL count after 96 hours (Data represent mean ± SEM. *n* = 5 per group. *P ≤ 0.05, **P < 0.01, ***P < 0.001. 1way ANOVA Dunnett‘s Multiple Comparison Test).

In a next step, we set out to define the optimal therapeutic dose of OZ for local pulmonary application in mice which is well tolerated but effectively inhibits the proteasome in the lung. We instilled increasing doses of 0.5 mg, 1 mg, or 5 mg per kg body weight of OZ intratracheally into the lungs of mice. OZ was suspended in 0.1% Pluronic F-127, a well-tolerated, FDA approved, biodegradable copolymer surfactant which has been shown to be non-toxic in epithelial cells [33–35]. Animals were sacrificed 24 or 96 hours after OZ application to analyse the CT-L proteasome activity of whole lung tissue as a measure of efficient proteasome inhibition and to determine cell counts in the bronchoalveolar lavage (BAL) as a read-out for acute lung injury (Fig 4A).

24 hours after instillation, we observed no significant reduction of proteasome activity with any of the OZ doses applied (Fig 4B) but the amount of polymorphonuclear leukocytes (PMNs) in the BAL increased up to 30% at the highest dose of 5 mg indicating an acute inflammatory response in the airspace of the lungs (Fig 4C). Proteasome activity was, however, significantly decreased in lungs after 96 hours of 0.5 mg or 1 mg/kg of OZ application (Fig 4D). The acute response declined and there was no indication of inflammatory PMN accumulation with these doses after 96 hours of instillation as determined by BAL cell count (Fig 4E). For further experiments, we thus chose a concentration of 1 mg OZ per kg body weight as an optimal non-harmful dose, which effectively inhibits proteasomes in the lungs after local application by intratracheal instillation.

### Evaluation of therapeutic effects of oprozomib after local application in a mouse model of lung fibrosis

**Fig 5:**
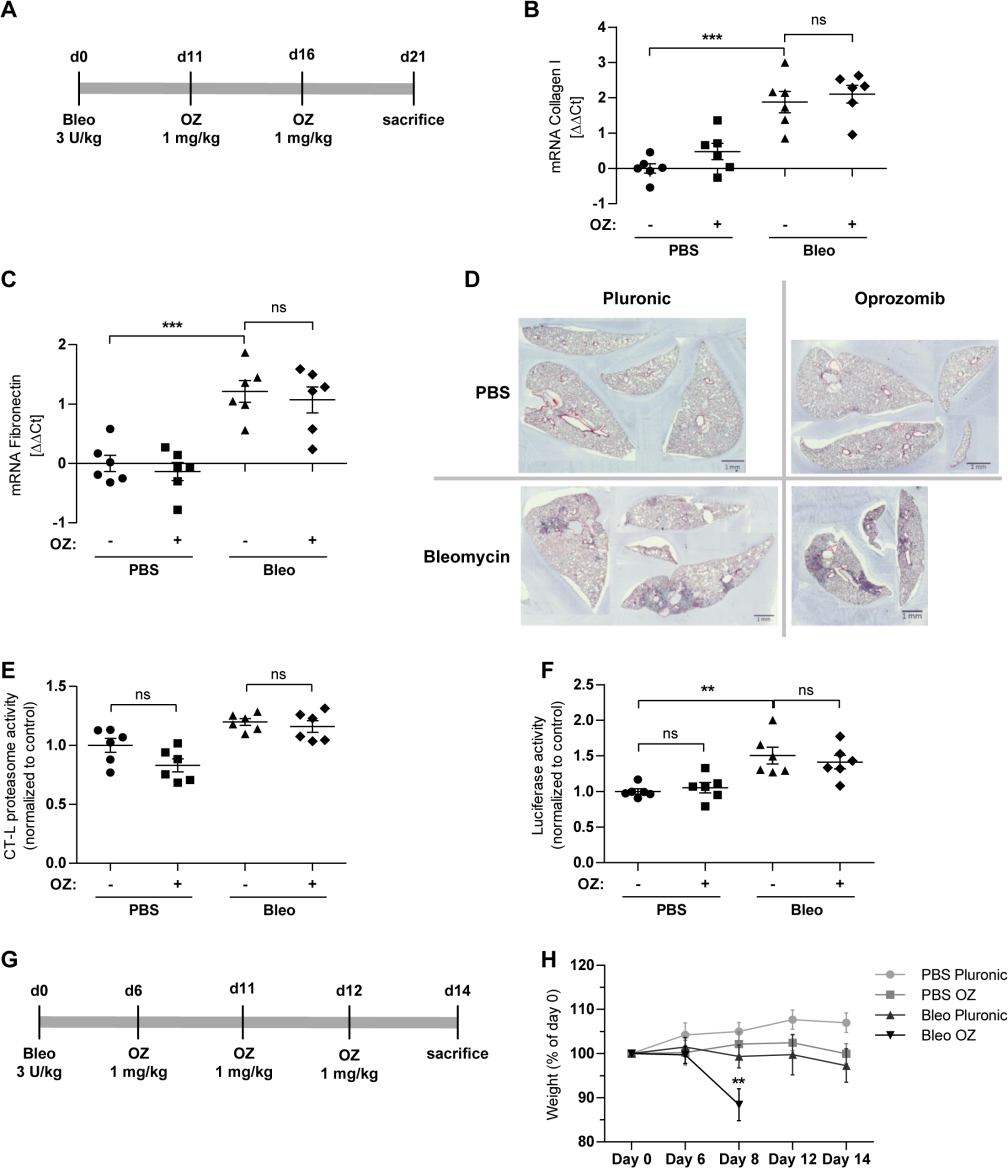
Local pulmonary application of oprozomib does not provide antifibrotic effects in the bleomycin mouse model. (A) Treatment scheme: local pulmonary application of OZ. (B) and (C) mRNA levels of Coll-I and fibronectin (Fn) (Data represent mean ± SEM. *n* = 6 per group. *P ≤ 0.05, **P < 0.01, ***P < 0.001. 1way ANOVA Bonferroni‘s Multiple Comparison Test). (D) H&E staining of lung slices. (E) CT-L proteasome activity and (F) luciferase activity of whole lung tissue. (G) Treatment scheme: repeated local pulmonary application of OZ. (H) Weight loss of animals at different time points (Data represent mean ± SEM. *n* = 6 per group. *P ≤ 0.05, **P < 0.01, ***P < 0.001. 1way ANOVA Bonferroni‘s Multiple Comparison Test).

We then investigated the therapeutic potential of locally applied OZ for the treatment of bleomycin induced lung fibrosis in mice. Here, we used the above described ODD-luc proteasome reporter mouse model to monitor the degree of proteasome inhibition by accumulation of the ODD-luc reporter in the mouse lungs together with assessment of the therapeutic effects of oprozomib. FVB-ODD luc mice were first challenged with bleomycin (3 U/kg) by intratracheal instillation. OZ (1 mg/kg of body weight) was applied 11 and 16 days after bleomycin challenge and mice were sacrificed at day 21 (Fig 5A). Of note, treatment of bleomycin challenged mice with OZ did not counteract bleomycin induced expression of the fibrotic marker genes collagen-I and fibronectin (Fig 5B and 5C). In accordance, H&E staining also did not show any therapeutic effects on lung fibrosis in response to OZ treatment (Fig 5D). We observed a slight but not significant decrease of the CT-L proteasome activity in whole lung tissue of control mice that had been treated with OZ only. Inhibition of the proteasome, however, was not detected in bleomycin-treated animals (Fig 5E). The luciferase reporter, supposed to accumulate upon reduction of proteasome activity, did not give any indication of reduced proteasome activity. Rather, we observed a significant increase in luciferase activity in lungs of bleomycin challenged mice compared to controls indicating an unexpected accumulation of the luciferase reporter in fibrotic lungs (Fig 5F).

Collectively, these results indicated that the ODD-luc reporter mouse strain is not a suitable model for monitoring proteasome inhibition in the fibrotic lungs of the bleomycin model as the reporter accumulated independently of any proteasome inhibition upon fibrotic remodelling of the lungs. In addition, the applied OZ treatment scheme did not effectively inhibit proteasomal activity in the lungs and we did not observe protective effects with regard to lung fibrosis. For these reasons and in view of the described resistance of FVB mice to develop liver fibrosis [36], we changed the mouse strain to C57BL/6 mice in our next animal experiments and used a 14 days bleomycin mouse model as this model is well established in our lab [27]. In addition, we increased the number of local OZ instillations to obtain more sustained local proteasome inhibition in the lung. In the next set of experiments, animals were treated at day 6, 8, and 12 after bleomycin challenge and sacrificed at day 14 (Fig 5G). While bleomycin or OZ treatment alone was well tolerated, the double challenge resulted in severe weight loss of all animals of this group so that the experiment had to be aborted for ethical reasons at this point (Fig 5H). These data suggest that local application of oprozomib to diseased and fibrotic lungs may even be fatal and that the therapeutic window for antifibrotic therapy by proteasome inhibitors is narrow.

### Evaluation of therapeutic effects of oprozomib after systemic application in a mouse model of lung fibrosis

**Fig 6:**
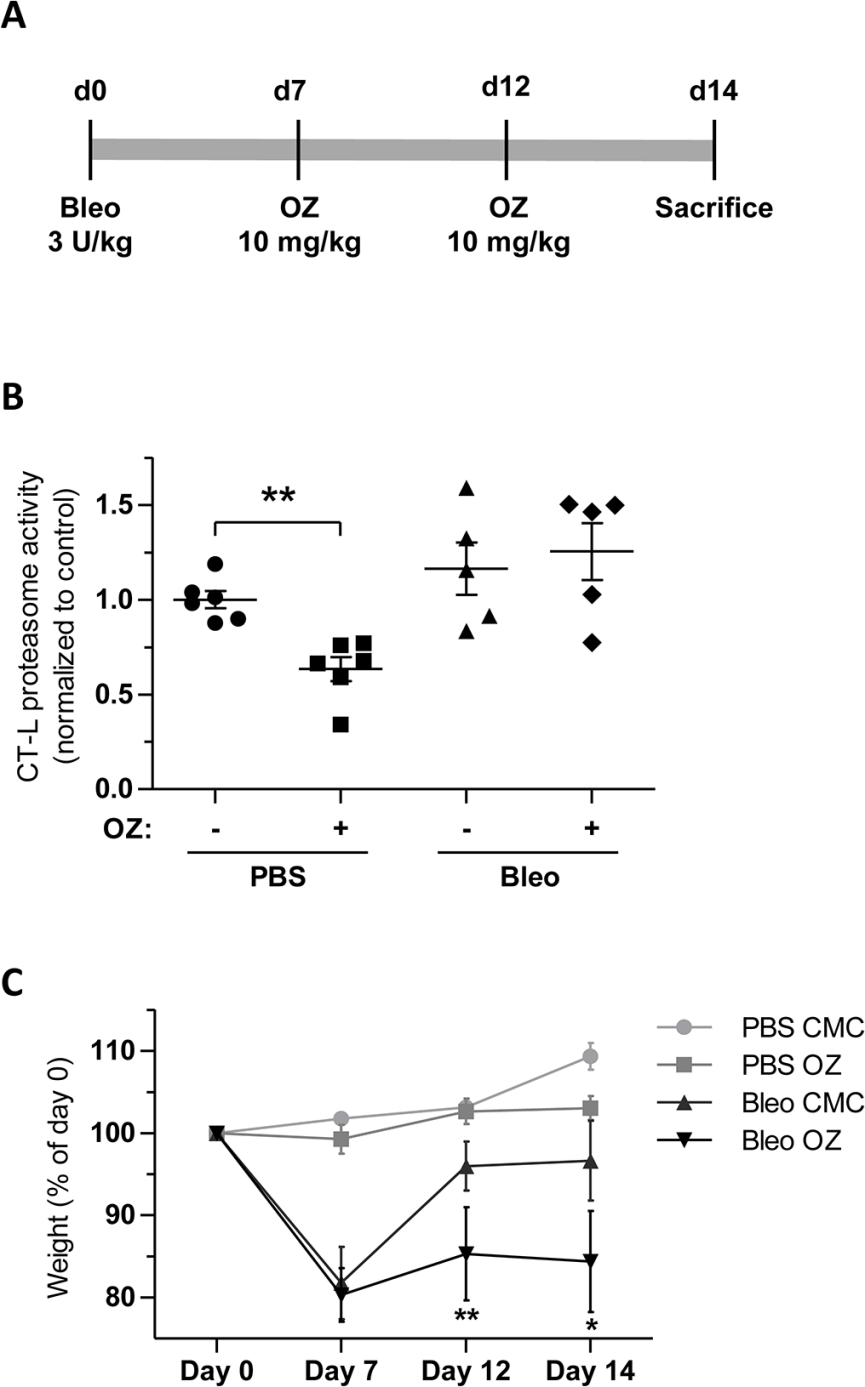
Oral application of oprozomib does not reduce proteasome activity in fibrotic lungs and is not well tolerated in bleomycin challenged animals. (A) Treatment scheme: repeated oral application of OZ. (B) CT-L proteasome activity (Data represent mean ± SEM. *n* = 5–6 per group. *P ≤ 0.05, **P < 0.01, ***P < 0.001. Mann Whitney t-test) and (B) weight loss of animals at different time points (Data represent mean ± SEM. *n* = 5–6 per group. *P ≤ 0.05, **P < 0.01, ***P < 0.001. 1way ANOVA Bonferroni‘s Multiple Comparison Test).

**Fig 7:**
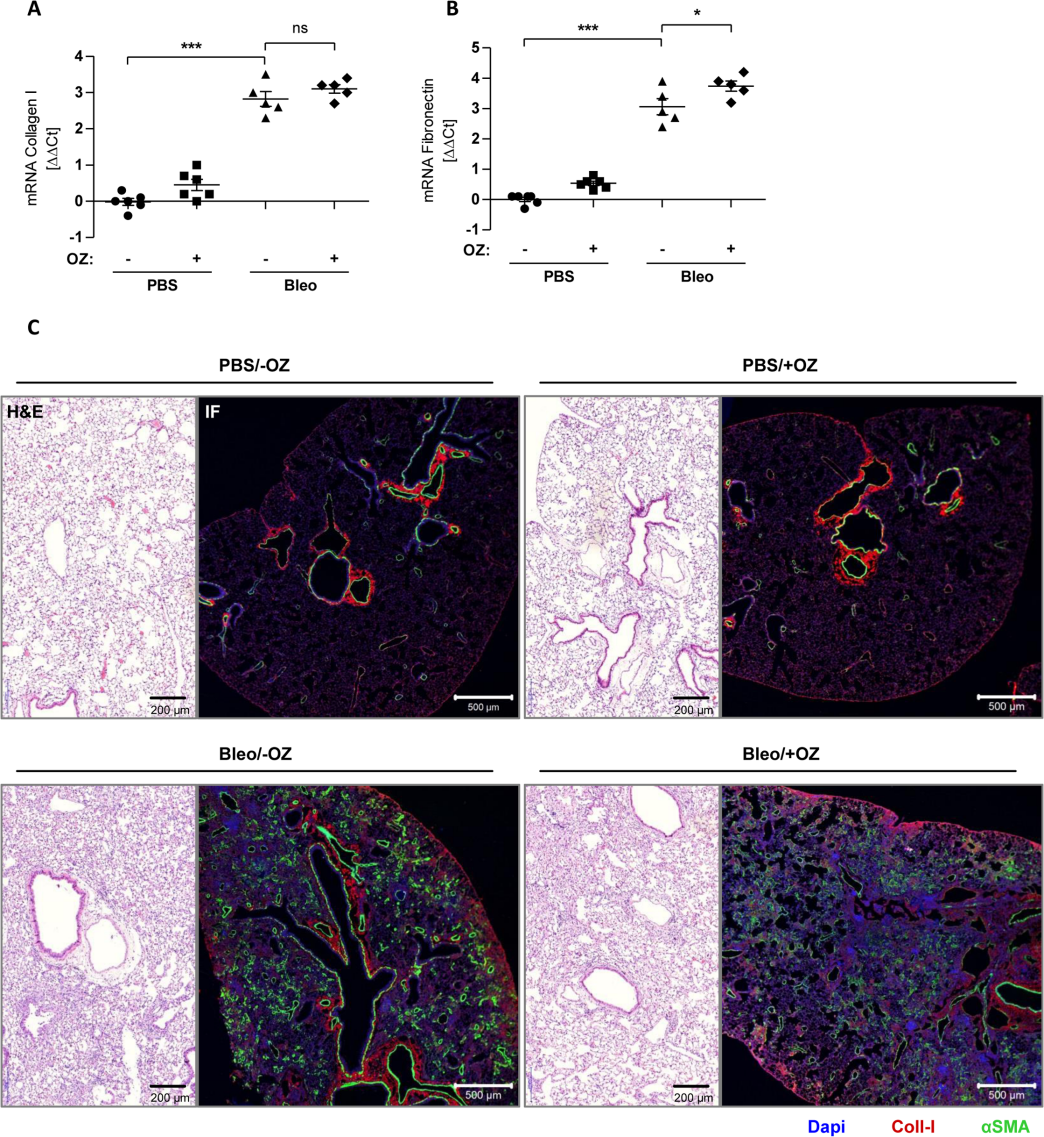
Oral application of oprozomib provides no antifibrotic therapeutic effects. (A) and (B) mRNA levels of Coll-I and Fn (Data represent mean ± SEM. *n* = 5–6 per group. *P ≤ 0.05, **P < 0.01, ***P < 0.001. 1way ANOVA Bonferroni‘s Multiple Comparison Test). (C) H&E staining of lung slices and immunofluorescence (IF) staining for Coll-I (red) and αSMA (green).

In our final approach, we tested, whether OZ might be better tolerated in the diseased lung after systemic application rather than local delivery to the lungs. An initial dose-finding experiment identified a concentration of 10 mg/kg body weight as an optimal dose of OZ that was well tolerated after repeated oral application in bleomycin challenged animals (data not shown). For oral application, OZ was suspended in 1% carboxymethylcellulose (CMC) and applied via a gavage needle 7 and 12 days after bleomycin treatment in female C57BL/6 mice. Animals were then sacrificed at day 14 (Fig 6A). OZ significantly reduced CT-L proteasome activity in healthy mouse lungs. This reduction was, however, not observed in fibrotic mouse lungs (Fig 6B). Oral OZ treatment alone did not show any toxic effects but co-treatment with bleomycin resulted in significant reduction of body weight (Fig 6C) similar to previous results after local application. mRNA expression of collagen-I and fibronectin was not altered by therapeutic OZ treatment compared to the bleomycin control group. Indeed, fibronectin mRNA was even increased in OZ treated fibrotic lungs (Fig 7A and 7B). We performed H&E staining to compare structural changes of the lung and immunofluorescence staining for the fibrotic markers Coll-I and αSMA (Fig 7C). However, we did not observe any antifibrotic effects of OZ in bleomycin challenged animals confirming our mRNA data of this experiment. Taken together, we were not able to show any therapeutic effects of OZ in the bleomycin mouse model. Indeed, we actually provide evidence for increased toxicity and a reduced therapeutic window by OZ in diseased animals.

## Discussion

In this study, we comprehensively analyzed inhibition profiles and antifibrotic effects of the novel 2^nd^ generation proteasome inhibitor oprozomib and investigated its therapeutic potential in the bleomycin mouse model after pulmonary or oral application.

In the human A549 cell line and in primary mouse ATII cells, OZ was less toxic than BZ. The NOAEL (No Observed Adverse Effect Level) of OZ exceeded that of BZ by a factor of 10, and OZ provided high selectivity for the chymotrypsin-like active site, whereas BZ inhibited also the caspase-like active site. It has been shown before that toxicities of proteasome inhibitors strongly depend on their inhibition profile [24]. Inhibition studies with selective inhibitors of the CT-L active site revealed that maximal toxicity in myeloma cells was only achieved by co-inhibition of the CT-L activity with one of the other two catalytic sites of the proteasome [37,38]. These data indicate that efficient inhibition of more than one active site is required for inducing cell death [24]. Although OZ has been shown to induce apoptosis in different multiple myeloma and cancer cell lines at doses similar to the ones applied here, unselective co-inhibition of the T-L and C-L active sites at toxic doses cannot be ruled out in these studies [17,18,39].

Inhibition profile and cytotoxicity of OZ were very similar in primary mouse lung fibroblasts compared to A549 cells. These fibroblasts were isolated from FVB-Luc reporter mice, in which the ODD-luc reporter is supposed to accumulate upon proteasome inhibition. Therefore, inhibition of the proteasome can also be monitored by the increase of luciferase activity. Only efficient inhibition of about 90% of the CT-L proteasome activity showed an effect on the reporter, which was rather weak with an increase of about 2 fold only. In contrast, previous studies reported a pronounced increase of luciferase activity of more than 14 fold after proteasome inhibition with BZ or MG132 in ODD-luc transfected HCT116 or Hela cells [32,40]. As MG132 and BZ both inhibit two active sites of the proteasome, it might be possible that proteasomal degradation of ODD-luc is not solely dependent on an active CT-L activity but also depends on the T-L and/or C-L activities of the proteasome. Such active site specific effects on substrate degradation have been shown before *in vitro* [41]. Furthermore, direct transfection of cells with the reporter construct might lead to higher expression levels of the reporter than in the cells of transgenic animals as used here.

OZ treatment of primary lung fibroblasts showed antifibrotic effects as indicated by dose dependent reduction in proliferation and Coll-I expression. In addition, OZ counteracted TGF-β induced expression of Coll-I and αSMA. Similar effects on lung fibroblasts have been shown by Mutlu et al. after treatment with a comparable dose of 200 nM of BZ [21]. Our results are in line with several studies that used fibroblasts of different tissue origins such as skin [19], heart [20], or kidney [42], and observed antifibrotic effects after treatment with inhibitors of the 20S core particle. Therefore, it can be assumed that partial and non-toxic inhibition of the 20S core particle in fibroblasts generally results in reduced proliferation and expression of profibrotic markers. However, we would like to emphasize that antifibrotic effects have only been shown for multi-catalytic-site inhibitors of the proteasome [19–21,43].

The underlying mechanism of this antifibrotic action is not well understood but several studies highlight the interaction with the TGF-β pathway [22,44]. Mutlu et al for example observed an increase of PPARγ in TGF-β treated primary human lung fibroblasts after BZ challenge and described antifibrotic effects by repression of SMAD mediated transcription through PPARγ [21]. Another study showed upregulation of the transcriptional repressor SnoN, which attenuated the transcriptional activity of TGF-β-induced R-Smad/Smad4 complexes [42]. However, given the pleiotropic effects of proteasome inhibition on cellular signalling molecules and transcriptional activators, narrowing the antifibrotic effects of proteasome inhibitors down to a single signalling pathway might be oversimplified. We have previously proposed that non-toxic inhibition of the proteasome induces a protective stress response in cells irrespective of the tissue origin which confers cell cycle arrest, overall attenuation of transcriptional regulation, and protection from stress [45,46]. Application of high-throughput techniques might provide a more comprehensive view on the cellular changes that mediate the observed antifibrotic effects.

In our study, we initially applied OZ locally into the lungs of mice to reduce systemic side effects and to increase local drug absorption. Indeed, we were able to reduce proteasome activity in the lungs of healthy mice after local application in a well-tolerated dose range. Pulmonary application of OZ in bleomycin challenged mice, however, was not well tolerated, especially when animals were treated three times with OZ. Moreover, we did not observe decreased pulmonary proteasome activities in response to OZ treatment, suggesting that proteasome inhibitors are either not effectively inhibiting the proteasome in the fibrotic lungs or that a compensatory increase in proteasome activity counteracts any inhibitory effects. Any attempt to obtain a more efficient inhibition of the proteasome in the lung by repeated OZ treatment even worsened lung damage. This observation is in line with studies by Fineschi et al., where treatment with proteasome inhibitors did not attenuate bleomycin induced lung fibrosis. Instead, bleomycin challenged animals which were systemically treated every 3–4 days with 0.8 mg bortezomib per kg body weight displayed reduced survival [23]. Very similar, we were unable to observe any antifibrotic therapeutic effects when we systemically applied OZ by oral application to reduce potential local toxicity of OZ after instillation into the lungs. Proteasome activity in the lung was significantly reduced by OZ treatment in healthy lungs but not in damaged and fibrotic lungs. Treatment of bleomycin challenged animals with OZ rather led to increased weight loss and reduced survival.

It also has to be considered whether the bleomycin mouse model is an appropriate model for IPF-related pulmonary fibrosis and therapeutic testing of drugs like proteasome inhibitors. Bleomycin initially causes acute lung injury and inflammation followed by fibrotic tissue remodelling in a very short time of about 7 to 9 days after intratracheal instillation. Within the fibrotic phase it resembles some of the histological patterns also seen in IPF such as increased expression of collagen and fibronectin and fibrotic remodelling. However, fibrotic remodelling in the bleomycin mouse model is reversible and therefore does not fully reflect the slow and irreversible progression of fibrosis as seen in IPF [47–49]. Therapeutic intervention with proteasome inhibitors in the beginning of the fibrotic remodelling phase might interfere with normal tissue repair and therefore even accelerate the damaging effects of bleomycin in this mouse model. Beside these limitations, the bleomycin mouse model remains the best investigated and probably most convenient model so far to test novel therapeutic compounds for pulmonary fibrosis [48].

Together with the published data [23], our results thus strongly point towards a very narrow therapeutic window of proteasome inhibitors for the treatment of pulmonary fibrosis. The therapeutic window might even be narrower for irreversible proteasome inhibitors such as OZ. Together with the observation that treatment of bleomycin challenged mice with proteasome inhibitors during the fibrotic remodelling phase even aggravated lung damage it is well feasible that functional proteasomes are even required for the fibrotic wound healing response in the lung. The challenge then would be to specifically target activated proteasome complexes in the fibrotic lung to the right degree and at the right time point.

## Supporting Information

S1 Fig Toxicity of oprozomib in primary mouse lung fibroblasts. (TIF)
