# Supplementary figures and images for: Validation of the 2nd Generation Proteasome Inhibitor Oprozomib for Local Therapy of Pulmonary Fibrosis
Source: PLoS One. 2015 Sep 4;10(9):e0136188. doi: 10.1371/journal.pone.0136188 (PMC4560391; doi:10.1371/journal.pone.0136188)

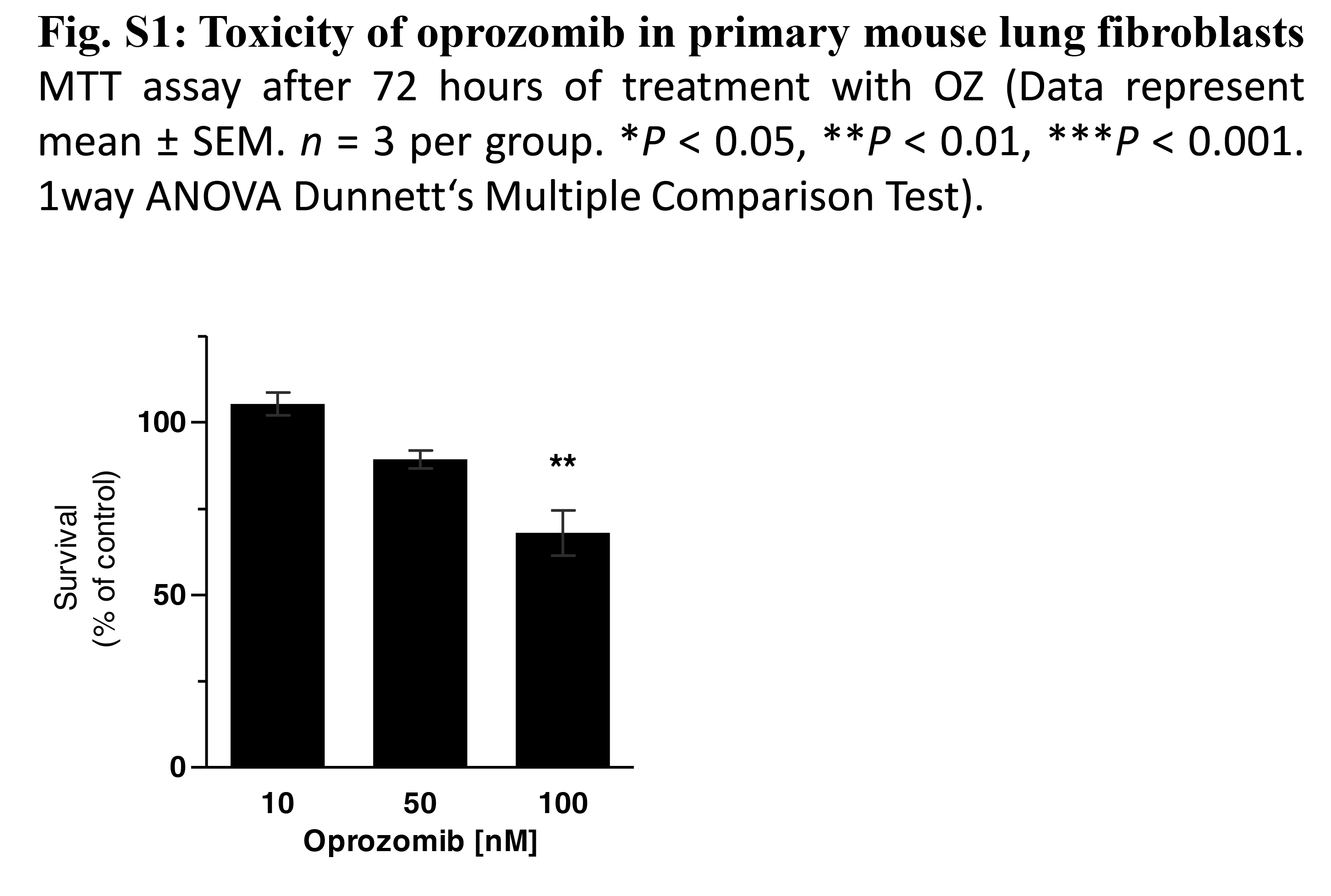

Supplement: S1 Fig — (TIF) [file pone.0136188.s001.tif]
